# EPuL: An Enhanced Positive-Unlabeled Learning Algorithm for the Prediction of Pupylation Sites

**DOI:** 10.3390/molecules22091463

**Published:** 2017-09-05

**Authors:** Xuanguo Nan, Lingling Bao, Xiaosa Zhao, Xiaowei Zhao, Arun Kumar Sangaiah, Gai-Ge Wang, Zhiqiang Ma

**Affiliations:** 1 School of Information Science and Technology, Northeast Normal University, Changchun 130117, China; biocs_nenu@126.com (X.N.); baoll601@nenu.edu.cn (L.B.); zhaoxs686@nenu.edu.cn (X.Z.); zhaoxw303@nenu.edu.cn (X.Z.); 2 School of Computing Science and Engineering, VIT University, Vellore 632014, Tamil Nadu, India; arunkumarsangaiah@gmail.com; 3 School of Computer Science and Technology, Jiangsu Normal University, Xuzhou 221116, China

**Keywords:** positive-unlabeled learning algorithm, pupylation sites, prediction, web server, support vector machine

## Abstract

Protein pupylation is a type of post-translation modification, which plays a crucial role in cellular function of bacterial organisms in prokaryotes. To have a better insight of the mechanisms underlying pupylation an initial, but important, step is to identify pupylation sites. To date, several computational methods have been established for the prediction of pupylation sites which usually artificially design the negative samples using the verified pupylation proteins to train the classifiers. However, if this process is not properly done it can affect the performance of the final predictor dramatically. In this work, different from previous computational methods, we proposed an enhanced positive-unlabeled learning algorithm (EPuL) to the pupylation site prediction problem, which uses only positive and unlabeled samples. Firstly, we separate the training dataset into the positive dataset and the unlabeled dataset which contains the remaining non-annotated lysine residues. Then, the EPuL algorithm is utilized to select the reliably negative initial dataset and then iteratively pick out the non-pupylation sites. The performance of the proposed method was measured with an accuracy of 90.24%, an Area Under Curve (AUC) of 0.93 and an MCC of 0.81 by 10-fold cross-validation. A user-friendly web server for predicting pupylation sites was developed and was freely available at http://59.73.198.144:8080/EPuL.

## 1. Introduction 

Prokaryotic ubiquitin-like proteins (Pup) are the first identified post-translational small modifier in prokaryotes [1,2]. They are disordered proteins, including 64 amino acids and an important signal for the protein’s selective degradation [3]. Pup usually attaches to substrate lysine via isopeptide bonds, and this process is called pupylation. Although the function of pupylation and ubiquitylation is similar, the enzymology participating in these processes is not the same [4]. Ubiquitylation requires three types of enzymes, including activating enzymes, ligases, and conjugating enzymes [5,6,7]. Pupylation only requires two types of enzymes (proteasome accessory factor A and deamidase of Pup).

Accurate identification of pupylation sites is an essential first step to better understand the underlying mechanism of protein pupylation. Though some large-scale proteomics technologies have been adopted to find the pupylation sites, they are usually time-consuming and laborious, especially for large-scale protein samples. Thus, computational methods were needed to effectively and accurately identify the potential pupylation sites in protein sequences. Lin et al. [8] developed the first pupylation site predictor, named GPS-PUP, the GPS means Group-based Prediction System. Tung et al. [9] constructed a predictor, iPup, in which the composition of k-spaced amino acid pairs feature (CKSAAP) was used. Zhao et al. [10] created a predictive model with five features and adopted feature selection methods to find the optimal feature set. Chen et al. [11] proposed a predictor, PupPred, which is based on the SVM and some sequence-derived features. Hasem et al. [12] introduced a profile-based CKSAAP to encode the pupylation sites and built a predictor called pbPUP. Wand et al. [13] employed the non-annotated lysine sites as unlabeled training samples and then used a two-class SVM to expand reliable negative set at each iteration. More recently, Jiang et al. [14] applied the positive-unlabeled learning technique to the prediction of pupylation sites, which combined the SVM and CKSAAP to construct the predictor PUL-PUP.

However, most of these computational methods artificially constructed the negative samples which included all the remaining non-annotated lysine residues. This negative samples dataset may contain some pupylation sites which were not validated. Then the classifiers trained on the experimentally-verified positive samples and such negative samples may be problematic and biased, and the final prediction performance was unsatisfactory. In this paper, we proposed an enhanced positive unlabeled learning algorithm to identify pupylation sites, EPuL, which enhanced the reliability of initial negative samples and then iteratively identified the non-pupylation sites from the unlabeled samples. Experimental results showed that our method achieved better performance when compared with other existing methods. Meanwhile, a user-friendly webserver of our proposed predictor was freely accessible at reference [15].

## 2. Results and Discussion

### 2.1. The Development of EPuL

The training dataset consisted of two kinds of subsets: (1) the positive dataset *P* and (2) the unlabeled dataset *U*. Positive-unlabeled learning has been used in bioinformatics and obtains satisfactory performance [16,17,18]. In this study, we proposed an enhanced positive-unlabeled learning algorithm called EPuL to predict pupylation sites. The detailed process of the algorithm is described as follows (Stage 1 is our proposed part. Stage 2 and Stage 3 are the same as PUL-PUP [14]):

**Stage 1:** Select the reliably negative initial set

The reliably negative dataset *RN* is initialized to an empty set and we use a vector $V_{s_{i}}$ to represent each sample in *P* and *U* by using the CKSAAP encoding scheme. By summing up all the vectors in *P*, we built the ‘positive representative vector (*pr*)’ and normalized it by using the formula below:

(1) $$pr=\sum_{i}^{\left|P\right|}V_{s_{i}}/|P|$$

Then, maximum distance rule is adopted, and the Euclidean distance was utilized to compute the average distance of each sample *s_i_* in *U* to *pr*:

(2) $$Avg\_dist+=\sum_{i}^{\left|U\right|}dist(pr,V_{s_{i}})/|U|$$

For each sample *s_i_* in *U*, the likely initial negative set *LN* was selected from *U* by *Avg_dist*; that is, if dist(*pr*,$V_{s_{i}}$) is more than *Avg_dist ×* ∂ (∂ = 1.05), we regard *s_i_* as a likely negative sample and put it into *LN*: *LN* = *LN* ∪ {$s_{i}$}.

To select the reliably negative initial set *RN*^0^ and enhance the reliability of *RN*^0^, we randomly divide *LN* into five likely negative subsets and each of them builds a model with *P*, which is based on the SVM. Subsequently, the remaining dataset *U* − *RN* is classified by the five models, respectively.

The common sequences *cs* which are predicted by five models and the negative support vectors *N_sv_* of the five models are all used to represent the reliably negative initial set *RN*^0^, in which, *RN*^0^ = *cs + N_sv_*.

**Stage 2:** Expand the reliably negative set

After the selection of reliably negative initial set, the reliable negative set was expanded by iteratively adding the negative examples from *U* using a series of two-class SVM classifiers. Specifically, at the *i*th iteration, the SVM classifier *f^i^* is firstly trained using dataset *P + RN^i^*; then, *f^i^* was used to classify the *U^i^* and each sample *x^i^* in *U^i^*, and each sample was obtained a decision value *f*(*x^i^*). To insure the reliability, samples belonging to the negative set need to satisfy:

$$f(x^{i})\leq T$$

Here, we set *T* = −0.50.

To overcome the problem of imbalance at each iteration, the negative support vectors $N_{sv}^{i}$ and the newly-predicted negative samples $N_{pred}^{i}$ are used to represent the existing negative set *RN^i^*, and we control the size of $N_{pred}^{i}$ less than 2 × |*P*|. Then, at the *i* + 1th iteration, $U^{i+1}=U^{i}{-N}_{pred}^{i}$; ${RN}^{i+1}=N_{pred}^{i}\cup N_{sv}^{i}$. Classifier *f^i+1^* is trained on *P* and current reliable negative training set *RN^i+1^*. 

With the expansion of negative set, the size of the remaining unlabeled set becomes less and less. Thus, iteration should be terminated at some point. When the number of the remaining unlabeled sets goes below the threshold 5 × |*P*|, the unlabeled data with the positive data would correspond to the maximum MCC.

**Stage 3:** Return the final classifier

After the extraction of the reliably negative set, a final SVM classifier is trained on *P* and the reliable negative set *RN*.

Algorithm 1 summarizes the detailed procedures of the proposed method EPuL.

**Algorithm 1.** An enhanced positive-unlabeled learning algorithm.Input*P*—Positive training set; *U*—Unlabeled training set; ∂—The distance coefficient; $V_{s_{i}}$—Sequence $s_{i}$ in *P* and *U*;*Model_1,2,3,4,5_*—Five models trained by five subsets with *P* respectively;*N_1,2,3,4,5_*—Five negative sets predicted by *Model_1,2,3,4,5_* on the remaining unlabeled training set respectively; cs—Common sequences of five negative sets *N_1,2,3,4,5_* $N_{sv}$—Negative support vectors of five *Model_1,2,3,4,5_*Output*F*—Final classifier.Stage 0:Initialization
*l*←*0*; *Avg_dist = 0*; *LN =* ∅; *RN =* ∅; *i*Stage 1:Select the reliably negative initial set
*pr* = $\overset{\left|P\right|}{\underset{i}{\sum }}V_{s_{i}}/|P|$; 
*Avg_dist* + = $\sum_{i}^{\left|U\right|}dist(pr,V_{s_{i}})/|U|$;
FOR
*i* from 1 to |*U*|
IF
*dist*(*pr*,$V_{s_{i}}$*)* > *Avg_dist* * ∂
*LN* = *LN*∪{*S_i_*};END IF
END FOR
Randomly divide the
*LN* into five subsets *D_1_*, *D_2_*, *D_3_*, *D_4_*, *D_5_*.
FOR
*i* from 1 to 5
*Model_i_* = SVM(*P*, *D_i_*); *N_i_* = *Model_i_*(*U* − *LN*);
END FOR
The common sequence are represented to reliably negative initial set *cs* = *N_1_* ∩ *N*_2_ ∩ *N*_3_ ∩ *N*_4_ ∩ *N*_5_; *RN*^0^ = *RN*^0^ ∪ *cs*; then the negative support vectors $N_{sv}$ of five models are included in ${RN}^{0}$ = ${RN}^{0}\cup^{\;}N_{sv}$.Stage 2Expand the reliably negative set
WHILE TRUE
IF
*U^l^* > 5∗*|P|*
$U^{l+1}$ = $U^{l}$−$N_{pred}^{l}$;
${RN}^{l+1}$ = $N_{pred}^{l}\cup^{\;}N_{sv}^{l}$;
ELSE IF
*U^l^* < 5 ∗ *|P|*
Go to Stage 3
END IF
Train a SVM classifier
*f^l^*^+1^ on the *P*∪*RN^l^*^+1^ with optimal parameter *C* and *γ*.
Each sequence
*x_i_* in *U^l^*^+1^ would have a decision value *f*(*x_i_*) through the obtained *f^l^*^+1^, use the threshold T to get the reliably negative set.
*l*
←
*l* + 1Stage 3Return the final classifier
Return
*F* = (*P*, *RN*)

### 2.2. The Performance of EPuL on the Training Dataset

**Figure 1:**
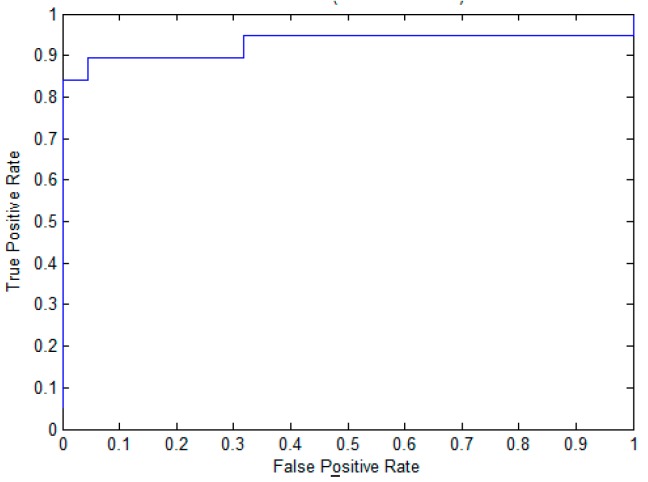
The ROC curve of EPuL on the training dataset.

To evaluate the effectiveness of the proposed method for pupylation site prediction, we compare EPuL with other methods, including PUL-PUP [14], PSoL [13], and SVM balance on the training dataset. In PSoL [13] algorithms, a two-class SVM is applied to filter the negative set from the non-annotated lysine sites and expand the negative set at each iteration. Additionally, in PUL-PUP [14] algorithms, the non-annotated lysine sites are treated as unlabeled samples and positive-unlabeled learning technique is used to predict of pupylation sites. The difference for us is on the selection of the initial negative set. As for SVM_balance, the negative training dataset is randomly selected from the non-annotated lysine sites. The ratio of the positive and negative training datasets is 1:1, which can avoid the imbalanced problem. The 10-fold cross-validation is performed on the positive set *P* and the reliably negative set *RN*, the results are shown in Table 1. We can see from Table 1 that EPuL yielded the best performance, a Sn of 84.21%, Sp of 95.45%, ACC of 90.24, and MCC of 0.81. EPuL achieves an improvement on the training dataset. Among this, the results of PSoL and SVM_balance are taken from PUL-PUP. To further demonstrate the superiority of EPuL, we also draw the ROC curve, as shown in Figure 1.

### 2.3. The Performance Evaluation on the Independent Testing Dataset

In order to further evaluate the performance of the proposed predictor, the independent testing dataset was utilized, which was completely blind to the training dataset. Table 2 presents the comparison of the results among EPuL, PUL-PUP, PSoL, and SVM-balance. Although SVM_balance can avoid the imbalanced problem, its prediction performance was the lowest, because the negative set of SVM_balance is randomly selected and are not the reliably negative samples. The PUL-PUP, which also uses the positive-unlabeled learning technique, mainly improves the performances through containing more information in *RN* at each iteration. However, the performance of PUL-PUP was not better than EPuL because the contained points are only based on the distance and not very precise. Especially, the stage 2 of EPuL is similar to PSoL, but we select the reliably negative initial set at stage 1, enhancing the positive-unlabeled learning at the beginning which would contribute to the selection of a more accurate negative set and make our algorithm more effective than PSoL.

We also compare our method with four existing predictors: iPUP, GPS-PUP, pbPUP, and PUL-PUP. We predefined three thresholds according to the SVM scores; that is, high (0.9672), medium (0.4032), and low (0.1088). Table 3 presents the detailed prediction performances on the independent testing dataset. The performance of our algorithm outperforms the existing predictors. For example, at the threshold low, the MCC of EPuL is 0.24, which is higher than that of GPS-PUP with an MCC of 0.1, iPUP with MCC of 0.15, pbPUP with MCC of 0.07, and PUL-PUP with MCC of 0.23. Moreover, our method obtains the best AUC value (0.78). Our classifier is iteratively trained on *P* and *RN*. Only with the reliable initial negative set can was obtain a more reliable negative set in the subsequent iterations. Thus, our method is more accurate and suitable for predicting pupylation sites than other methods.

### 2.4. Feature Analysis

**Figure 2:**
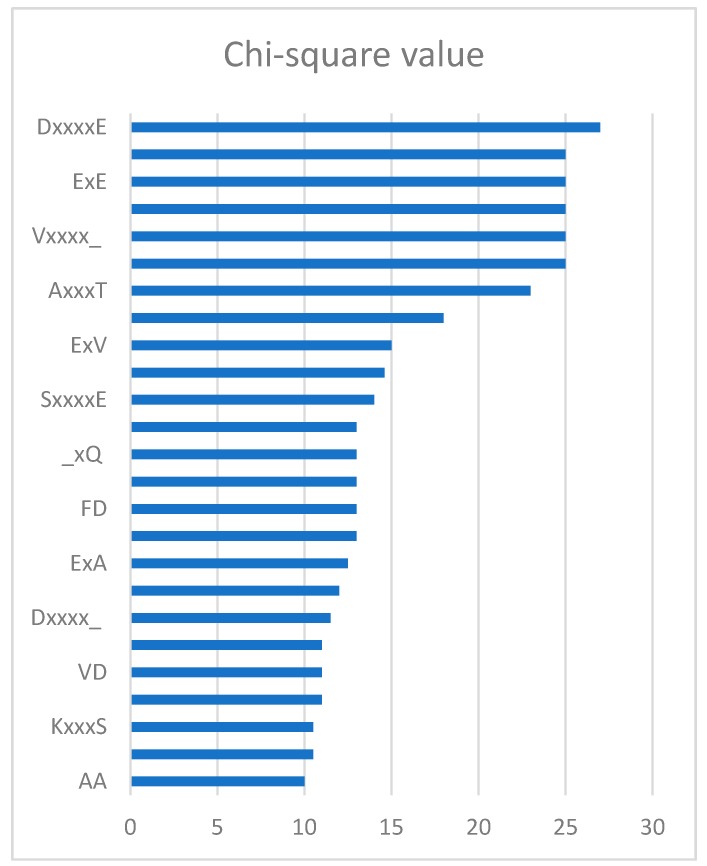
Top 25 *k*-spaced amino acid pairs.

**Figure 3:**
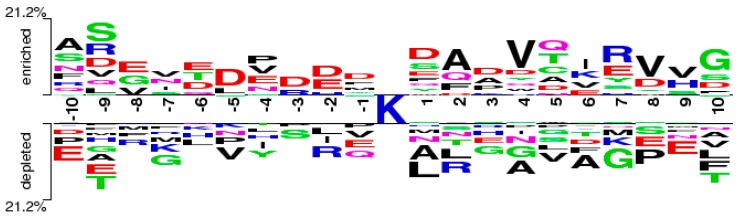
The two-sample-logos of the composition of *k*-spaced amino acid pairs surrounding the pupylation site and non-pupylation site.

Through the feature selection method, we can find the ranked features generated by the CKSAAP encoding scheme in Figure 2. The importance of these features was also clearly and intuitively shown in Figure 3. For example, the feature ExE which represents the EE residue pair spaced by any amino acid, is enriched in the positive pair and not in the negative pair. From Figure 2, we can see that the features frequently appeared in the top 25 amino acid pairs, which also frequently occurred in Figure 3.

### 2.5. Case Study

To further verify the generalization of our model, we adopted EPuL for a total of 1116 pupylated proteins, which are identified by high-throughput proteomics methods [19] and have unknown pupylation sites. Among the total proteins, EPuL successfully identified 2102, 3265, and 3899 pupylation sites at the threshold of ‘high’, ‘medium’ and ‘low’, respectively. The result of the predicted pupylation sites is available in Supplementary File 1.

## 3. Materials and Methods

### 3.1. Datasets

In this paper, the training dataset and the independent testing dataset of iPup [9] were used. The training dataset included 162 proteins, which consisted of 183 experimentally-validated pupylation sites and 2258 artificial generated non-annotated pupylation sites. The former were regarded as positive samples, and the latter were regarded as unlabeled samples. The independent testing dataset included 20 proteins, including 29 experimentally-verified pupylation sites and 408 non-annotated pupylation sites. Though the independent testing dataset was highly imbalanced, it can reflect the real effectiveness of different methods. Similar to the current pupylation site prediction methods [8,9,10,11,12,13,14], the sliding window method was adopted to encode each sample in the dataset. The window size was set to 21 here, in accordance with [9].

### 3.2. Construction of Feature Vectors

In this study, the composition of the *k*-spaced amino acid pairs (CKSAAP)-based encoding scheme was applied to encode each sample. CKSAAP could show the association of the residues surrounding pupylation sites and it has been successfully applied to other kinds of PTM site prediction problems [20,21,22]. Taking *k* = 0 as an example, for a sequence fragment including 2n + 1 amino acids, there are 441 0-spaced residue pairs (i.e., AA, AC,…). Then a 441-dimensional feature vector can be defined as:

$${\left(\frac{N_{AA}}{N_{total}},\;\frac{N_{AC}}{N_{total}},\;\ldots \ldots .,\;\frac{N_{\_\_}}{N_{total}}\right)}_{441}$$

The value of each component is the probability of each amino acid pair. When there are *n* AA pairs in the sequence fragment, the value of *N_total_* is 441 for any window size, and the value of $\frac{n}{N_{total}}$ is the probability of the corresponding AA pair.

With the increase of *k*, the accuracy and the sensitivity increase, while the computational complexity and the required time also increase. In this paper, the value of parameter *k* in CKSAAP was set to 0, 1, 2, 3, and 4. Thus, each sample is represented by 2205 dimension features. For example, for the pair A and D, the *k*-spaced amino acid pairs for *k* = 0, 1, 2, 3, and 4 are represented as AD, AxD, AxxD, AxxxD, AxxxxD.

### 3.3. Feature Selection

In order to remove the irrelevant and redundant features, we utilized the chi-square test and sequential backward feature elimination algorithm, which was the same as iPUP [9]. Each feature would have a value by chi-square test and sequential backward feature elimination algorithm was used to select optimal feature subset. Firstly, we ranked the 2205 dimension features according to the value of chi-square. Then, we iteratively removed 10 features with the lowest value in a sequential backward feature elimination algorithm. Finally, the feature subset with the highest performance was used as the optimal feature subset to train the model. Figure 2 shows the top 25 CKSAAP features ranked by using the chi-square test and we used the top 150 features as the optimal feature subset, which was the same as iPUP [9]. The complete list of the optimal feature subset is shown in Supplementary File 2.

### 3.4. Support Vector Machine

A support vector machine with the kernel radial basis function (RBF) was the core learning algorithm of EPuL. The LibSVM [23] package widely used in the area of bioinformatics [24,25,26] was used to train the final prediction model. A grid search strategy based on 10-fold cross-validation was utilized to find the optimal parameters.

### 3.5. Performance Evaluation of EPuL

Five measurements were employed to evaluate the performance of our proposed predictor [21]. These measurements included sensitivity (SN), specificity (SP), accuracy (ACC), and Matthews’ correlation coefficient (MCC). These measurements are defined as the following formulas:

$$SN=\frac{TP}{TP+FN}$$

$$SP=\frac{TN}{TN+FP}$$

$$ACC=\frac{TP+TN}{TP+TN+FP+FN}$$

$$MCC=\frac{TP\;\times TN\;-FN\;\times FP}{\sqrt{\left(TP+FN\right)\times \left(TP+FP\right)\times \left(TN+FP\right)\times \left(TN+FN\right)}}$$

where *TP*, *FP*, *TN*, and *FN* denote the number of true positives, false positives, true negatives, and false negatives, respectively. Matthews’ correlation coefficient (*MCC*) provides an overall performance of binary classification.

## 4. Conclusions

In this paper, we proposed a new predictor, EPuL, to identify the protein pupylation sites. We aim to make the initial selected negative set reliable, and then a more and more reliable negative set will be selected in later iterations. As this process continues, the final negative set will be as reliable as possible. The proposed enhanced positive-unlabeled learning algorithm outperforms the existing predictors. Moreover, the most likely pupylation and non-pupylation sites can be predicted in non-annotated lysine sites by using EPuL. We are confident that the proposed method could also be applied in the identification of other types of PTMs sites. A user-friendly web server is freely available at reference [15]. In our future research, except for the predictor EPuL proposed in this paper, we will use some state-of-the-art metaheuristic algorithms to identify the protein pupylation sites, such as monarch butterfly optimization (MBO) [27], earthworm optimization algorithm (EWA) [28], elephant herding optimization (EHO) [29], moth search (MS) algorithm [30], and krill herd (KH) [31,32,33,34,35].

## Reference

## Figures and Tables

**Table 1:** Ten-fold cross-validation performance of EPuL, PUL-PUP, PSoL and SVM_balance.

Method	Sn (%)	Sp (%)	ACC (%)	MCC	AUC
EPuL	84.21	95.45	90.24	0.81	0.93
PUL-PUP	82.24	91.57	88.92	0.74	0.92
PSoL	67.50	73.60	70.55	0.42	0.80
SVM_balance	76.71	63.65	69.88	0.40	0.77

**Table 2:** Independent test performance of EPuL, PUL-PUP, and PSoL.

Method	Sn (%)	Sp (%)	ACC (%)	MCC	AUC
EPuL	72.41	71.57	71.63	0.24	0.78
PUL-PUP	68.97	70.83	70.71	0.22	0.77
PSoL	51.72	73.14	71.62	0.13	0.74
SVM-balance	62.07	67.4	67.05	0.15	0.7

**Table 3:** The performance of EPuL and four exiting pupylation sites predictors on the independent testing dataset.

Predictors	Thresholds	Sn (%)	Sp (%)	ACC (%)	MCC	AUC
GPS-PUP	High	31.03	89.46	85.62	0.16	
	Medium	34.48	85.54	82.19	0.14	0.6
	Low	41.38	76.72	74.43	0.1	
iPUP	High	48.28	82.84	80.55	0.2	
	Medium	51.72	76.47	74.83	0.16	0.66
	Low	55.17	72.06	70.94	0.15	
pbPUP	High	17.24	88.48	83.75	0.04	
	Medium	31.03	80.15	76.89	0.07	0.6
	Low	41.38	69.85	67.96	0.07	
PUL-PUP	High	51.72	83.33	81.24	0.22	
	Medium	65.52	76.72	75.97	0.24	0.77
	Low	68.97	72.79	72.54	0.23	
EPuL	High	37.93	89.46	86.04	0.21	
	Medium	58.62	79.90	78.49	0.23	0.78
	Low	68.97	74.02	73.68	0.24	

## Supplementary Materials

The following are available online. Supplementary File 1: The result of the predicted pupylation sites, Supplementary File 2: The complete list of the optimal feature subset.
