# Supplementary material for: EPuL: An Enhanced Positive-Unlabeled Learning Algorithm for the Prediction of Pupylation Sites
Source: Molecules. 2017 Sep 5;22(9):1463. doi: 10.3390/molecules22091463 (PMC6151806; doi:10.3390/molecules22091463)
Supplement: Supplementary file 1 [file molecules-22-01463-s001.pdf]

Top 150 k-spaced amino acid pairs

DxxxxE	LxK	FxxK	MxxxxE	WxV	LC
VxxxxR	DxD	MxxxxK	MxxxxA	HxN	VxxC
ExE	GxxxY	SxxxxS	MxxE	TxxM	HY
VxxN	VxxxxS	ExxxxH	CG	HxxxT	FxxxH
Vxxxx_	IxP	PxxxxA	AxxxxN	KC	GxC
Vxxx_	ExxxxL	VN	RxxxM	DxxxxH	PxxW
AxxxT	AxxxxD	VxxY	FxxxN	VxxxC	SxxxW
VxD	FxxD	GxxxxR	MxxT	ExC	IxxxW
ExV	NI	TxxxL	PxxQ	NxxQ	NxxxM
LE	AxxxE	DxxI	QY	TxxW	PxxC
SxxxxE	KP	GxM	QxxxxP	MxxxP	CxK
ExxxK	LxG	HxxxK	HxxxI	NxxxH	ExR
_xQ	YxxxK	HxG	QxxxN	PxxxxH	ExL
T_	KxxxxA	MxxxK	FxxF	CA	CxxE
FD	KxK	IxxxxI	WxxA	CxxA	ExN
VV	IS	YxP	IxxM	FxxxY	TxL
ExA	GxxK	GxxxM	HxxxS	CxxxxK	ExM
DxxxR	YK	TxxxH	VxxxW	CxxxxA	TxR
Dxxxx_	NxxG	KxM	PxxM	TxxxxW	ExK
DxxF	LK	HD	AC	CxL	ExP
VD	MxxxA	RxH	TxM	CxA	ExQ
LxxA	MxE	YxxY	MxxI	CV	ExG
KxxxS	TxK	PM	_xxxA	CxxxI	ExT
ExxxxE	LxxP	MxG	RxxxxH	IxxxxM	CxxQ
AA	IxH	PxxF	MxxP	NxM	CxxP
